# External Validation of Prediction Models for Pneumonia in Primary Care Patients with Lower Respiratory Tract Infection: An Individual Patient Data Meta-Analysis

**DOI:** 10.1371/journal.pone.0149895

**Published:** 2016-02-26

**Authors:** Alwin Schierenberg, Margaretha C. Minnaard, Rogier M. Hopstaken, Alma C. van de Pol, Berna D. L. Broekhuizen, Niek J. de Wit, Johannes B. Reitsma, Saskia F. van Vugt, Aleida W. Graffelman, Hasse Melbye, Timothy H. Rainer, Johann Steurer, Anette Holm, Ralph Gonzales, Geert-Jan Dinant, Joris A. H. de Groot, Theo J. M. Verheij

**Affiliations:** 1 Julius Center for Health Sciences and Primary Care, University Medical Center Utrecht, Utrecht, the Netherlands; 2 Saltro Diagnostic Center for Primary Care, Utrecht, the Netherlands; 3 Department of Public Health and Primary Care, Leiden University Medical Center, Leiden, the Netherlands; 4 Department of Community Medicine, University of Tromsø, Tromsø, Norway; 5 Chinese University of Hong Kong, Hong Kong, China; 6 Horten Centre for Patient Oriented Research and Knowledge Transfer, University Zurich, Zurich, Switzerland; 7 Department of Infectious Diseases, Odense University Hospital, Odense, Denmark; 8 Division of General Internal Medicine, University of California San Francisco, San Francisco, United States of America; 9 Department of Family Medicine, CAPHRI School for Public Health and Primary Care, Maastricht University Medical Centre, Maastricht, The Netherlands; 10 Institute of Molecular and Experimental Medicine, Cardiff University, Cardiff, United Kingdom; Ohio State University College of Medicine, UNITED STATES

## Abstract

**Background:**

Pneumonia remains difficult to diagnose in primary care. Prediction models based on signs and symptoms (S&S) serve to minimize the diagnostic uncertainty. External validation of these models is essential before implementation into routine practice. In this study all published S&S models for prediction of pneumonia in primary care were externally validated in the individual patient data (IPD) of previously performed diagnostic studies.

**Methods and Findings:**

S&S models for diagnosing pneumonia in adults presenting to primary care with lower respiratory tract infection and IPD for validation were identified through a systematical search. Six prediction models and IPD of eight diagnostic studies (N total = 5308, prevalence pneumonia 12%) were included. Models were assessed on discrimination and calibration. Discrimination was measured using the pooled Area Under the Curve (AUC) and delta AUC, representing the performance of an individual model relative to the average dataset performance. Prediction models by van Vugt et al. and Heckerling et al. demonstrated the highest pooled AUC of 0.79 (95% CI 0.74–0.85) and 0.72 (0.68–0.76), respectively. Other models by Diehr et al., Singal et al., Melbye et al., and Hopstaken et al. demonstrated pooled AUCs of 0.65 (0.61–0.68), 0.64 (0.61–0.67), 0.56 (0.49–0.63) and 0.53 (0.5–0.56), respectively. A similar ranking was present based on the delta AUCs of the models. Calibration demonstrated close agreement of observed and predicted probabilities in the models by van Vugt et al. and Singal et al., other models lacked such correspondence. The absence of predictors in the IPD on dataset level hampered a systematical comparison of model performance and could be a limitation to the study.

**Conclusions:**

The model by van Vugt et al. demonstrated the highest discriminative accuracy coupled with reasonable to good calibration across the IPD of different study populations. This model is therefore the main candidate for primary care use.

## Introduction

Pneumonia is a major cause of death in developed countries [1,2] and requires clinical treatment, whereas other lower respiratory tract infections (LRTIs) such as acute bronchitis are self-limiting [3]. The accurate diagnosis of pneumonia by a general practitioner (GP) is therefore important, but challenging as the routine use of chest x-radiography (CXR) for all patients presenting with LRTI is not feasible. Consequently, GPs mainly rely on signs and symptoms (S&S) in the diagnosis of pneumonia.

Prediction models based on S&S have been proposed to decrease diagnostic uncertainty and prevent improper prescription of antibiotics and accompanying bacterial resistance [4–7]. Before considering the use of a prediction model in daily clinical practice, it is essential that its performance is empirically evaluated in datasets that were not used in the model development [8–10]**.** Such a study, in which the discrimination and calibration [11] of a prediction model are evaluated in new patients, is referred to as external validation [10,12]. Discrimination is the ability of the model to differentiate between diseased and non-diseased patients, whilst calibration signifies the agreement between predicted and observed probability of disease [12]. Evaluation of clinical usefulness with regard to improving patients outcomes or changing GP behavior are not part of external validation [13]. External validation is required to quantify optimism caused by model overfitting [14] or deficiencies in the statistical modeling during model development, such as incorrect handling of missing data or a small sample size. Validation is also important to assess the model’s transportability to other sites with arguably similar patients [9,12,15].

External validation of newly developed prediction models is rarely performed and generally of poor quality [13], but a necessary step before use in clinical care. Therefore, this type of study is receiving increasingly more attention and has a central role in the recently published reporting guideline for prediction research (TRIPOD statement [16] and S1 TRIPOD Checklist).

A limited number of external validation studies on diagnostic models or pneumonia have been performed [17–19], but none included patient data of the multiple study sites and recently developed models [19]. Therefore, a meta-analysis using individual patient data (IPD) from multiple studies was performed in order to extensively assess and compare the performance of all published S&S models for the diagnosis of pneumonia in primary care.

## Materials and Methods

### Selection of published models

Models eligible for inclusion were logistic regression models including S&S for predicting the probability of pneumonia in primary care patients with acute cough or suspected LRTI. Because of the cross-sectional nature of our study and our dichotomous outcome (pneumonia present or absent) we included only logistic regression models. These prediction models were identified through the following strategy: (a) screening references of the European Respiratory Society management guidelines for adults with LRTI [20]; (b) eligibility assessment of models included in previously published validation studies [17–19]; (c) systematically searching PubMed, EMBASE and the Cochrane Library, using the terms “pneumonia”, “LRTI”, “C-reactive protein (CRP)” and a diagnostic filter [21,22] (S1 Appendix, reference date: August 2012, 21^st^). CRP, an inflammation marker, was incorporated in the search for the purposes of a supplemental study on the added value of CRP over signs and symptoms alone [Minnaard MC et al. 2015. In revision for CMAJ], but is not further investigated in the current study. After the identification of all eligible models, experts in the field were asked to identify missing models.

### Selection of IPD for validation of published models

IPD for model validation was identified using the same systematical search in PubMed, EMBASE and the Cochrane Library as described above (S1 Appendix). Prospective studies were included when recording disease status of pneumonia and clinical S&S. Pneumonia status was included as a dichotomous variable (i.e. absent or present) and should have been determined by a physician using by CXR [23], CT or MRI imaging techniques. Individual studies were included when containing patients who: (a) were at least 18 years old; (b) presented trough self-referral in primary care, ambulatory care or at an emergency department with an acute or worsened cough (≤28 days of duration) or with a clinical presentation of LRTI; (c) consulted for the first time for this disease episode; (d) were immunocompetent.

### Methodological quality assessment of IPD

Two reviewers (AS, JG) independently assessed the characteristics and methodological quality of the included IPD using the QUADAS-2 [24] in order to identify potential sources of bias and improve the interpretation of results (S1 Table). IPD were compared to the original study report on the total number of patients and the frequencies of single variables for error checking. If necessary, authors were contacted for information on quality assessment criteria or when datasets showed unexpected missing or invalid values.

### Missing data

Missing values in IPD were regarded as missing at random (MAR). Single imputation was performed on individual dataset level [25] when missingness per IPD dataset did not exceed 33%. Predictors were considered absent when missingness exceeded 33% or when a predictor was not recorded entirely. Models could not be validated in IPD datasets containing absent predictors. This implies that the number of analyzed patients might differ between the models validated.

### Statistical analysis

The performance of included prediction models was assessed by discrimination and calibration. All performance measures were determined using the original models, without adjustment of model’s intercept and coefficients. This enables us to evaluate the performance of the various models, when applied directly in another setting, as is often done in practice, without updating or refitting the model to better accommodate the new setting.

Discrimination was quantified using the pooled Area Under the (ROC) Curve (AUC) and the deltaAUC. Pooled AUC was quantified by first calculating the AUC and 95% confidence interval (CI) for each model individually per IPD dataset, followed by combining the individual AUCs in a pooled AUC using inverse variance weighing [26,27]. This two-step approach ensures accurate estimation of the pooled AUC in account for potential heterogeneity in AUC estimates [28]. As the absolute value of discrimination may differ considerably between IPD datasets, model performance was subsequently evaluated on a relative scale, using the deltaAUC. The deltaAUC represents the difference in discriminative performance between an individual model (AUC) and the average performance of all models (mean AUC) within an IPD dataset. Calibration of included prediction models was assessed across different risk groups in each individual dataset. Risk groups with a low (0–10%) predicted risk of pneumonia, an intermediate risk (10–30%) and a high risk (30–100%) were defined. Per risk group the average predicted probability was calculated and compared to the proportion of pneumonia (i.e. the observed prevalence of pneumonia) in this group of patients. To obtain reliable estimates, the average probabilities were only calculated when at least 5 subjects per risk group could be included. In the case both a model and its development dataset were included in this study, the IPD of such a study was excluded from the external validation process. Data were analyzed with IBM SPSS statistics for Windows Version 20 (IBM Corp; Armonk, NY), R (v.2.15) including the “RMS” and “ROCR” packages for R [29] and Excel 2010 for Windows (Microsoft Inc; Redmond, Washington). A prospective study protocol was formulated, indicating the main study objectives of the IPD study and the general methods for the current external validation study (S1 Protocol).

The Institutional Review Board of the University Medical Center Utrecht was not consulted for this meta-analysis as the study used only anonymous data from previously performed studies for which both informed consent and ethical approval had already been obtained.

## Results

### Selection of models

After assessment of published studies validating S&S models [17–19] and the European Respiratory Society guideline [20,30], six pneumonia prediction models for primary care use were included [18,19,31–34]. No suitable additional models were identified neither through our systematic search, nor after inquiry with experts in the field. The prediction models included between three to six predictors, the most frequent being fever (in 5 models), crackles (in 4 models), coryza (in 3 models), cough, dyspnea, diminished breath sounds and tachycardia (in 2 models). The predictors asthma, duration of illness, chest pain, diarrhea, fever (symptom), myalgia, phlegm, sore throat, sweating and tachypnea were all included in one model (Table 1 and S2 Table). S3 Table presents the in- and exclusion criteria of all model development studies and studies contributing IPD.

**Table 1:** Overview of included prediction models to diagnose pneumonia in a primary care setting and their incorporated predictors.

Model	Total	Diehr et al. [31]	Singal et al. [34]	Heckerling et al. [18]	Melbye et al. [33]	Hopstaken et al. [32]	van Vugt et al. [19]
Total predictors in model		6	3	5	6	3	6
***History***							
Absence of asthma	1			•			
Duration of illness	1				•*		
***Symptoms***							
Chest pain	1				•		
Coryza (absence)	3	•			•		•
Cough (dry)	2		•			•	
Diarrhea	1					•	
Dyspnea	2				•		•
Fever	1				•*		
Myalgia	1	•					
Phlegm	1	•					
Sore throat	1				•		
Sweats (night)	1	•					
***Signs***							
Crackles	4		•	•	•		•
Diminished breath sounds	2			•			•
Fever	5	•	•	•		•	•
Tachycardia	2			•			•
Tachypnea	1	•					

• = predictor present

*combined predictor.

### Selection of IPD for validation of published models

**Fig 1:**
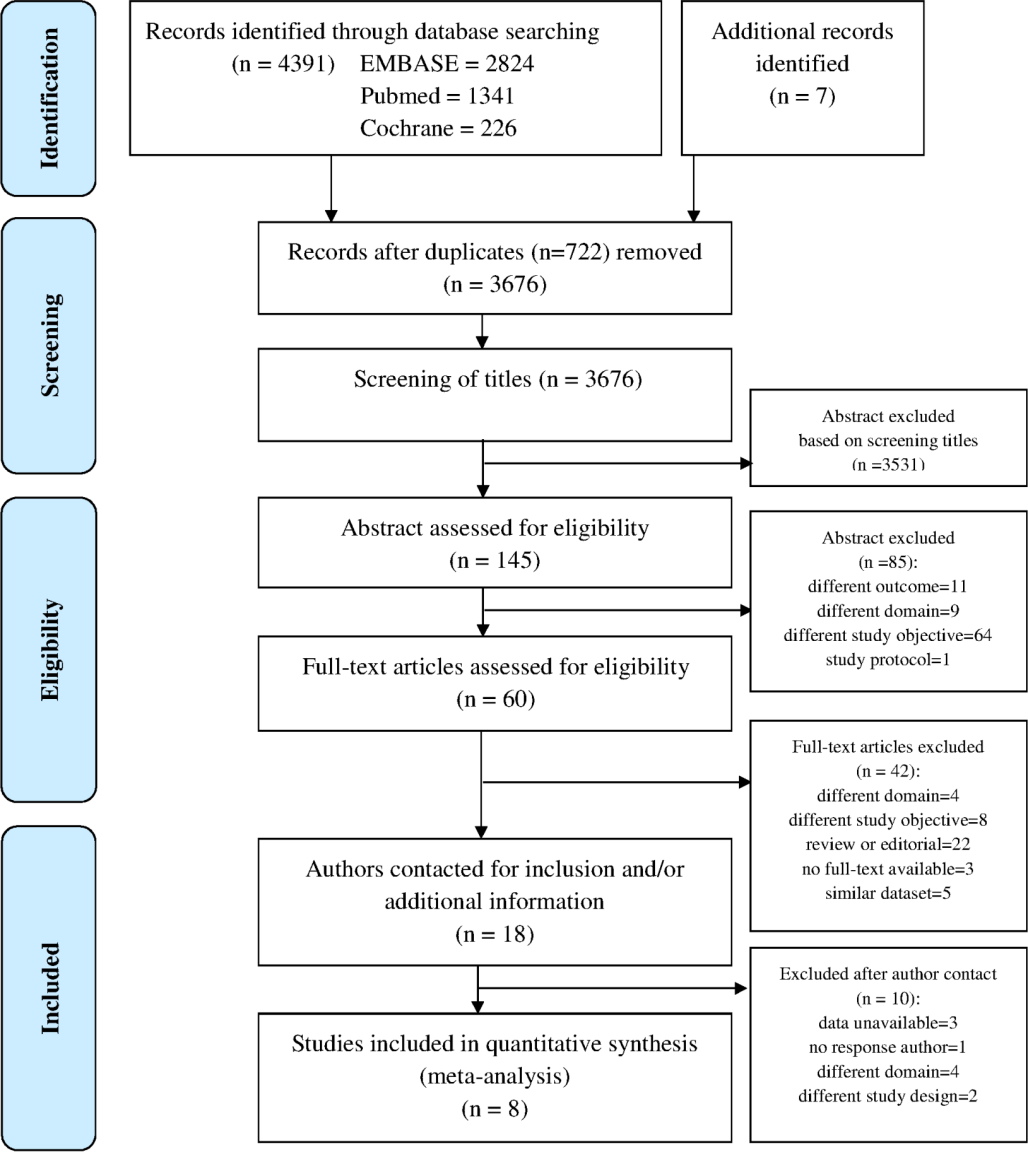
PRISMA flow diagram of the selection process of IPD used for external validation of prediction models [39].

Eighteen of the 3676 identified studies appeared eligible for inclusion. Authors of these eighteen studies were requested to provide additional information and original data. Six studies did not fit the inclusion criteria, one author did not respond to our request and three authors were unable to provide the original study data (Fig 1). Eventually, the IPD of eight studies (N = 5308) were included [17,19,32,33,35–38].

### Characteristics of IPD

Table 2 gives a detailed presentation of the baseline characteristics in all included IPD datasets. Of the eight included studies, five included patients visiting a GP [17,19,32,36,38], one included patients visiting a primary care out-of-hours service [33] and two studied self-referred patients to an emergency department [35,37] (Table 2). Of all IPD, 55% (N = 2820 patients) were contributed by the study by van Vugt et al. The mean age was 49 years (SD = 18) when taking all IPD patients together. The mean age of separate studies was lower in patients from Melbye et al. and Flanders et al., with a mean age of 33 (SD = 14) and 40 (SD = 16) years, respectively. In individual datasets the proportion of males varied between 40 and 50%. The prevalence of pneumonia ranged from 5% to 43%. In only one study providing IPD all predictors were present [35], in all other studies proving IPD one or more predictors were not recorded. If predictor were recorded the highest percentage of missing values per predictor never exceeded 33% (max. 28%). No dataset showed missing values for the outcome pneumonia. One of the included IPD datasets had previously been imputed using hot-deck imputation [35].

**Table 2:** Baseline characteristics of included individual patient datasets used in the external validation of prediction models for pneumonia in primary care setting (numbers are percentages [%] per dataset or specified otherwise).

Characteristic	Validation dataset								
	Melbye et al. [33]	Hopstaken et al. [32]	Flanders et al. [35]	Graffelman et al. [17]	Holm et al. [36]	Rainer et al. [37]	Steurer et al. [38]	van Vugt et al. [19]	All datasets
***Patient characteristics***									
Setting	OHD	GP	ED/AC	GP	GP	ED	GP, ED	GP	AC/ED/GP/OHD
Number of patients	402	243	168	129	364	561	621	2820	5308
Pneumonia	5%	13%	12%	20%	13%	43%	21%	5%	12%
Age, mean (SD)	33 (14)	52 (16)	40 (16)	50 (14)	50 (16)	53 (22)	47 (16)	50 (17)	49 (18)
Gender, Male	41%	47%	41%	47%	49%	53%	50%^1^	40%	44%
Duration illness in days, mean (SD)	10 (14)	Categorized^2^	7 (5)	9 (6)	--	17 (9)	7 (10)	10 (10)	8,4 (10)
Smoker	56%	33%	11%	36%	45%	17%	29%	28%	30%
Asthma	10%	19%	11%	6%	8%	--	--	10%	10%
***Symptoms***									
Cough	91%	92%	100%	98%	98%	88%	97%	100%	97%
Chest pain (lateral)	53%	60%	40%	23%	64%	40%	29%	46%	45%
Coryza	80%	38%	69%	59%	--	50%	--	71%	67%
Diarrhea	--	8%	14%	24%	--	9%	--	7%	8%
(Daily) Fever, subjective	31%	35%	59%	85%	42%	83%	56%	35%	47%
Dyspnea	69%	77%	51%	76%	72%	56%	36%	57%	57%
Myalgia	54%	62%	55%	59%	--	50%	--	50%	52%
Sore throat	73%	39%	65%	39%	--	50%	--	--	55%
Phlegm	88%	55%	55%	79%	81%	77%	49%	79%	75%
(Night) Sweats	84%	61%	58%	--	--	42%	--	--	60%
***Signs***									
Crackles	11%	21%	9%	60%	--	--	20%	9%	573
Diminished breath sounds	5%	--	17%	12%	--	--	12%	13%	13%
Heart rate, p.m. (SD)	79 (13)	--	85 (19)	82 (11)	81 (15)	98 (18)	--	77 (12)	81 (15)
Respiratory rate, p.m. (SD)	--	Categorized^3^	18 (4)	21 (4)	19 (4)	19 (3)	17 (6)	17 (4)	18 (4)
Temperature, C° (SD)	37.3 (0.7)	37.5 (0.8)	37.3 (0.8)	37.9 (0.7)	37.4 (0.6)	37.8 (1.1)	37.4 (1)	36.7 (0.6)	37.1 (1)

OHD = Out of Hours Department, GP = General Practitioner, ED = Emergency Department, AC = Ambulatory Clinic

^1^Data from original publication

^2^ Categorized as ≤2, 3–7, 8–28 days

^3^Categorized as >20 p.m.

"--" = Variable missing

### Methodological quality assessment of IPD

In general, the assessment of study quality of the included datasets raised little concern of bias (S1 Table). Nonetheless, four studies showed a risk of bias and/or applicability concerns in the patient selection [17,33,35,38]. Two studies presented potential bias concerning flow and timing [33,35], as the acquisition of the reference test was left up to the physician’s judgment (partial verification), which may have induced misclassification of pneumonia. To adjust for potential misclassification one of these two study performed the reference standard in a 25% random sample (showing no additional cases of pneumonia) [33]. Furthermore, in one IPD dataset the CXR results were missing; therefore the discharge diagnosis (primarily based on CXR results) was used in the meta-analysis to define pneumonia [37]. Moreover, this study reported a high prevalence of pneumonia (43%) [37], indicating a potential applicability concern in the patient selection for the purposes of this validation study.

### Performance of models in individual patient datasets

Each of the six included models could be externally validated in the IPD of at least three and up to seven datasets (Table 3); the model by Diehr et al. in three datasets (N = 972), Singal et al. in seven datasets (N = 4747), Heckerling et al. in four datasets (N = 3519), Melbye et al. in three datasets (N = 540), Hopstaken et al. in four datasets (N = 3678) and the model by van Vugt et al. in three datasets (N = 699).

**Table 3:** Discriminative performance of pneumonia prediction models per dataset, measured as Area Under the ROC Curve (AUC) and as pooled AUC in all suited individual patient data (IPD).

Model	Validation dataset								Development AUC (95% CI)	Pooled AUC (95% CI)^†^	Patients in IPD /development (N =)
	Melbye et al. [33]	Hopstaken et al. [32]	Flanders et al. [35]	Graffelman et al. [17]	Holm et al. [36]	Rainer et al. [37]	Steurer et al. [38]	van Vugt et al. [19]			
Van Vugt et al. [19]	0.78	X	0.89	0.60	X	X	X	D	0.70 (0.65–0.75)	0.79 (0.74–0.85)	699/2820
Heckerling et al. [18]	0.69	X	0.89	0.62	X	X	X	0.66	0.82 (0.78–0.86)	0.72 (0.68–0.76)	3519/1134
Diehr et al. [31]	X	0.57	0.76	X	X	0.64	X	X	NA	0.65 (0.61–0.68)	972/474
Singal et al. [34]	0.68	0.62	0.81	0.63	0.62	X	0.61	0.64	0.73 (0.69–0.77)	0.64 (0.61–0.67)	4747/255
Melbye et al. [33]	D	0.57	0.62	0.49	X	X	X	X	0.75 (0.66–0.84)	0.56 (0.49–0.63)	540/402
Hopstaken et al. [32]	X	D	0.58	0.61	X	0.52	X	0.56	0.70 (0.59–0.80*)	0.53 (0.50–0.56)	3678/243

X = Model not validated in dataset due to missing predictors, D = Development dataset (AUCs shown under “Development”), NA = Not available (none reported in development study)

* 95% CI not available in original study report (recalculated in original dataset)

^†^AUC of Development dataset (“D”) not included.

**Fig 2:**
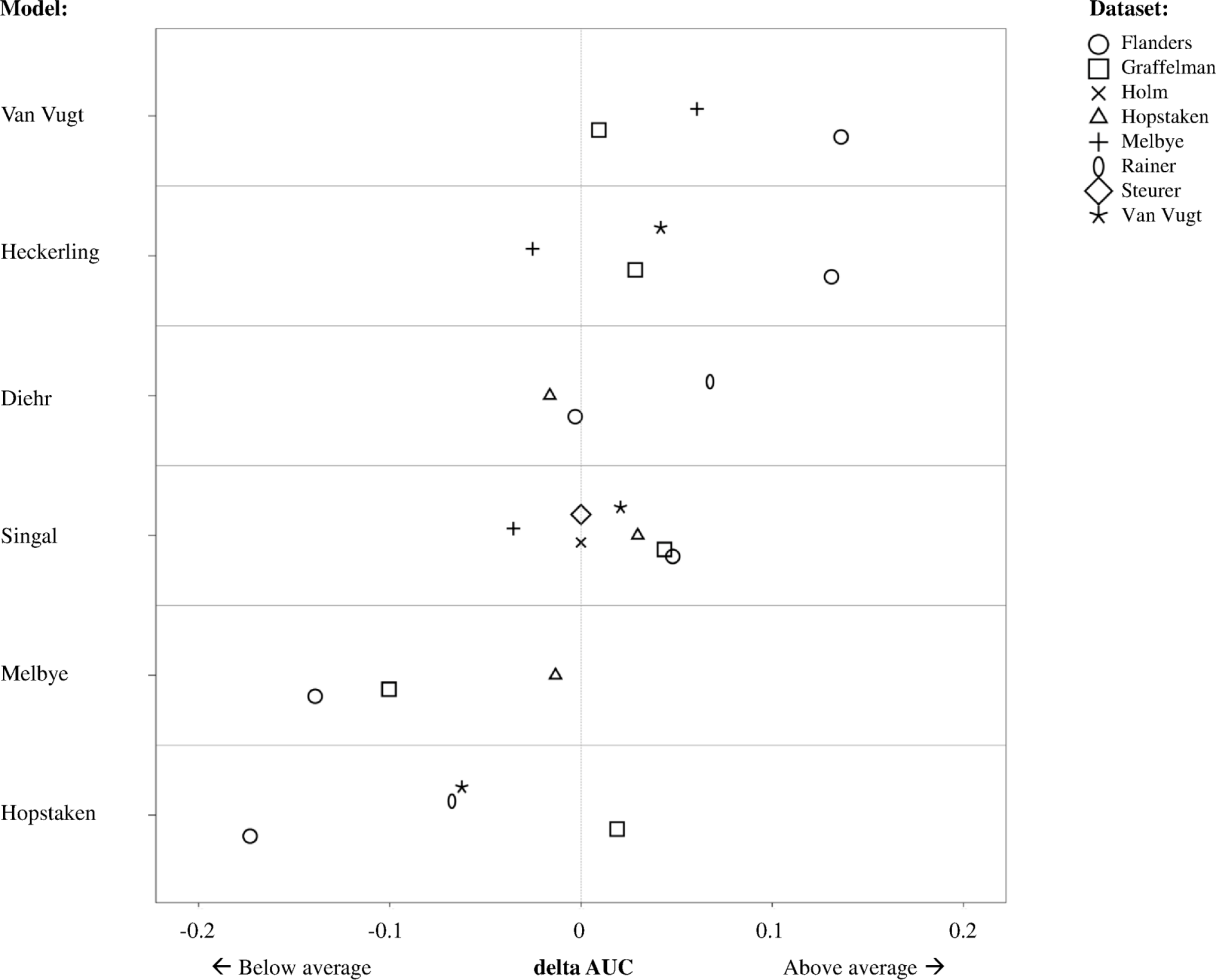
Graphic representation of model performance relative to dataset average AUC, measured as delta AUC. Each point represents the performance of an individual model relative to the average performance of all models per dataset (deltaAUC, calculated as individual model AUC minus [–] the mean AUC of dataset). The figure shows how the discriminative performance per model, in the datasets in which it could be validated, is compared to the discriminative performance of the other models in that same dataset. For example, we see that the model by van Vugt et al. performs above average in all datasets in which it could be validated (i.e. Graffelman et al., Melbye et al, and Flanders et al). Furthermore, by studying the figure more closely, we can see the order of what model performed best in what dataset. For example, the models by van Vugt et al. and Heckerling et al. perform best in the dataset by Flanders et al., followed by the models by Singal et al., Diehr et al., Melbye et al. and Hopstaken et al.

The model by van Vugt et al. demonstrated the highest pooled AUC of 0.79 (95% CI 0.74–0.85), compared to an AUC of 0.7 in the development study. The model by Heckerling et al. demonstrated a pooled AUC of 0.72 (95% CI 0.68–0.76, development AUC of 0.82), Diehr et al. of 0.65 (0.61–0.68, development not available), Singal et al. of 0.64 (0.61–0.67, development 0.73), Melbye et al. of 0.56 (0.49–0.63, development 0.75) and Hopstaken et al. of 0.53 (0.5–0.56, development 0.7). When evaluating the individual model performance relative to the average dataset performance, using the deltaAUC as measure, a similar ranking was demonstrated compared to the ranking based on the pooled AUC. The model by van Vugt et al. demonstrated a higher than average AUC in all datasets, with deltaAUCs ranging from 0.14 to 0.01 (Fig 2 and S4 Table), and was followed by Heckerling et al. (0.13 to -0.03), Diehr et al. (0.07 to -0.02), Singal et al. (0.05 to -0.04), Melbye et al. (-0.01 to -0.14) and Hopstaken et al. (0.02 to -0.17).

**Fig 3:**
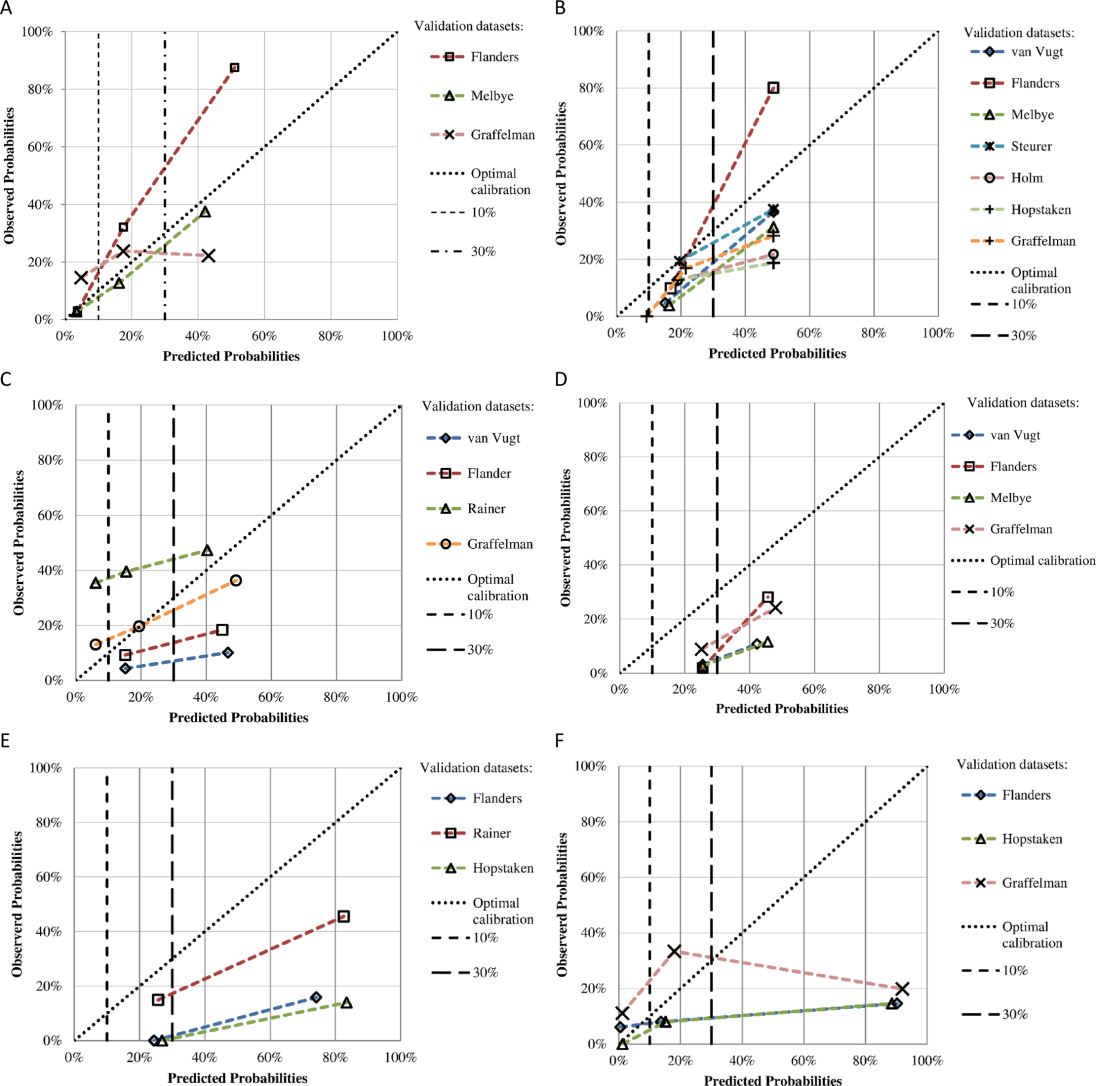
Calibration plots of prediction models clustered per risk group with low (0–10%), intermediate (10–30%) and high (30–100%) predicted probabilities. Calibration results are presented for each validation dataset where the model could be validated. Plots show how well the predicted probabilities (x-axis) agree with observed probabilities (y-axis). For perfect agreement, the calibration curve falls on the ideal diagonal line (optimal calibration). Two vertical cut-off lines for 10% and 30% risk of pneumonia are depicted. (A) Calibration plot of the model by van Vugt et al. (B) Calibration plot of the model by Singal et al. (C) Calibration plot of the model by Hopstaken et al. (D) Calibration plot of the model by Heckerling et al. (E) Calibration plot of the model by Diehr et al. (F) Calibration plot of the model by Melbye et al.

For each model [18,19,31–34], calibration curves were plotted by comparing the predicted probability to the observed probability in each individual dataset (Fig 3). The calibration plot of the model by van Vugt et al. demonstrated the closest agreement between the model’s predictions and the observed prevalence of pneumonia (Fig 3A). The model by Singal et al. lacked the potential to assign patients to a low risk of pneumonia, but showed a rather uniform prediction pattern in the other risk groups, where in general the model slightly overestimated the predicted probabilities (Fig 3B). The model by Hopstaken et al. showed a linear relation between the predicted probabilities and prevalence of pneumonia in all datasets. However, this relation varied considerably, from consistent overestimation in one dataset and an underestimation in another (Fig 3C). The models by Heckerling et al. and Diehr et al. demonstrated consistent overestimation of the predicted probabilities and lacked the potential to assign patients to a low risk of pneumonia (Fig 3D and 3E, respectively). The model by Melbye et al. lacked a clear linear relation between the observed probabilities and prevalence of pneumonia (Fig 3F). Pooling of calibration results was not possible due to heterogeneity of results and, therefore, not further pursued.

## Discussion

### Main findings

This study assessed discrimination (pooled AUC an deltaAUC) and calibration of six previously published primary care S&S models for patients with suspected LRTI in the IPD of eight diagnostic studies (N = 5308).

The model by van Vugt et al., demonstrated the highest pooled and relative discriminative performance, with a pooled AUC of 0.79 and deltaAUCs between 0.14 and 0.01. The model by Heckerling et al. followed with a pooled AUC of 0.73 and deltaAUCs between 0.13 and -0.03. The models by Diehr et al., Singal et al. Melbye et al. and Hopstaken et al., demonstrated lower average and relative discriminative performance, with respective pooled AUCs of 0.65, 0.64, 0.56 and 0.53, and marginally positive or negative deltaAUCs values. Calibration of the models by van Vugt et al. and Singal et al. was acceptable, demonstrating reasonable overall agreement in the predicted probabilities and presence of pneumonia, and allows for optimization with simple recalibrated methods. Calibration of the remaining models showed signs of overfitting or varying degrees of systematic over- or underestimation of predicted probabilities between different datasets, impeding simple recalibration.

### Interpretation of findings

It is common that performance of a prediction model decreases when validated in new patients. Such a decrease is typically caused by the difference in case-mix of, arguably similar, patients. However, when the decrease in performance is larger than expected other mechanisms could have caused overfitting of the model in the development study, such as a (too) small development dataset or a too elaborate selection of candidate predictors [14]. Furthermore, in some cases the replacement of absent predictors in the external validation data may have led to lower discriminative performance of the model, e.g. ‘dry cough’ in the model of Hopstaken et al. was only measured in a single dataset [17] and, therefore, the predictor ‘cough’ was used. The model by van Vugt et al. showed a better discrimination in external validation compared to the development study. This somewhat unusual finding might be caused by the partial verification of the disease status in two of the included datasets [33,35]. In both datasets CXR acquirement was dependent on physician judgment, whereas patients not receiving a CXR were considered healthy. Consequently, clinical information (e.g. signs and symptoms) could have influenced the disease status and lead to an overestimation of the discriminative performance of a prediction model [40]. However, it is likely that all models would equally benefit from potential overestimation of the discriminative performance in these two datasets and also be of little impact, as most models could be validated in these datasets.

Concern in performance differences would not have existed if all models would have been validated in all IPD. In our study such a comparison was not conceivable as in five of the included IPD dataset one or more required predictors were absent. To approach an equal comparison between models and minimize the performance differences we used the deltaAUC. Here both methods (pooled and deltaAUC) demonstrated similar results.

Performance between models could also be affected by the inclusion criteria used in a study contributing IPD. For example, when patients are selected on the basis of specific clinical characteristics (e.g. fever) one might expect that the performance of models including such variables (predictors) will be negatively influenced in a validation study [41]. However, the good performance of some of the included models, when evaluated in a mixed IPD population including patients with various likelihoods of pneumonia, indicates that they can be used beyond the first step of the diagnostic process.

In this study we performed a visual assessment of calibration in various clinically relevant risk groups. Per group it was assessed how the predicted risk of pneumonia compared to the true prevalence of pneumonia. In general, included models failed to assign extreme predictions (closer to 0 or 1), meaning it is challenging to completely rule out or prove the presence of pneumonia. Either such extreme predictions were not made at all by the model (e.g. for low risks <10%) or did not correspond well with the true prevalence of pneumonia (e.g. for higher predicted risks >30%). This phenomenon can be expected when presenting patients are in general reasonably healthy and when studying a clinically heterogeneous disease, like pneumonia, where disease course is influenced by a variety in airway pathogens and patient characteristics such as comorbidity and frailty. Future research should focus on the recalibration of original models to ensure the accurate predictions in all types of patient populations, while preserving discrimination [42]. However, in models lacking consistency in calibration (e.g. by overfitting), simple recalibration methods may not suffice. Two of the included models cases (Diehr et al. and Melbye et al.) included no intercept. This may be an explanation for the poor calibration of these models. In subsequent investigations it is recommended to add an intercept to improve performance of these models. However, such amendments were beyond the scope of this review.

Finally, although various reference standards were allowed to determine pneumonia status, all included studies diagnosed pneumonia using CXR. Arguably, the diagnostic properties found in the present analysis may be lower, or higher, when applied to settings where alternative reference standards for pneumonia than CXR are applied. However, as no consensus on a gold standard for pneumonia exists, none of the studies raised concern about the reference standard in the QUADAS-2 assessment and because we used the same outcome definition for both the included models as for the included datasets, we do not expect this to introduce bias (e.g. diagnostic or selection bias) in our study.

### Strengths and limitations

To our knowledge this IPD meta-analysis validated all primary care S&S prediction models for pneumonia in a large composite dataset of IPD of high quality diagnostic studies. Included models could be validated in at least three external data sources, providing reliable estimates of the pooled AUC. Nonetheless, it is important when comparing models to focus on results obtained within the same validation dataset, in a paired comparison using deltaAUCs, as the absolute value of discrimination differed between validation datasets. Calibration of models in multiple validation datasets is notoriously hard to quantify. Therefore, we created clinically relevant risk groups to detect potential weaknesses in calibration that can be translated to the clinical setting.

A potential limitation of this study was the use of alternative (definitions for) predictors when specific predictors from published models were missing (S2 Table). However, we only used these alternative predictors when sufficiently appropriate or when they could be calculated with the help of other predictors. Moreover, we presume that these types of predictors (e.g. “sweats” for “night sweats”) are often used in a similar and interchangeable fashion in daily practice and are therefore comparable. Even when alternative predictors were considered, the performance evaluation of several models was hampered due to absence of predictors. This complicates straightforward comparison of these models and could have theoretically induced bias in model performance. However, by assessment of the discriminative performance according to two different methods, which incorporated a within model comparison (i.e. deltaAUC) this evaluation was arguably justified.

Lastly, in our study the prevalence of pneumonia ranged between 5–43% in the included IPD datasets, which is generally higher than the prevalence of 6% typically found in a primary care setting [43]. The large variation in prevalence reflects both a variation in setting of included studies and a difference in the inclusion criteria applied in included studies. This may have led to the inclusion of IPD with a broad case-mix, ranging from patients with acute cough to suspected pneumonia. However, as the key purpose of an external validations study is to evaluate the performance of prediction models in other–but arguably comparable–patients, the heterogeneity in the IPD patient population due to differences in inclusion criteria does not interfere with the primary aim of our study.

## Conclusions

Prediction models can be of value for GPs by discriminating between patients with and without pneumonia but they fail to assign very high or low risks. Of all published primary care S&S models, the model by van Vugt et al. demonstrated the highest discriminative accuracy coupled with reasonable to good calibration in IPD of different study populations. This model is therefore the main candidate for use in primary care.

## Supporting Information

S1 TRIPOD Checklist TRIPOD Checklist for prediction model development and validation with added text excerpts. Some of the items were not applicable (NA) to the current study.
(PDF)

S1 Appendix Search strategies for PubMed, EMBASE and the Cochrane Library. (PDF)

S1 Protocol Diagnostic value of c-reactive protein for Pneumonia in primary care acute cough patients: An individual patient data meta-analysis. (PDF)

S1 Table Overview of methodological quality of included validation datasets according to QUADAS-2 assessment [24]. (PDF)

S2 Table Prediction rules of included diagnostic models for pneumonia in primary care. (PDF)

S3 Table In- and exclusion criteria of all model development studies and studies contributing IPD. (PDF)

S4 Table Relative discriminative performance of pneumonia prediction models within datasets, measured as delta AUC. Numbers depict the difference of individual model’s AUC to the average AUC of the dataset. (PDF)
