# Supplementary material for: External Validation of Prediction Models for Pneumonia in Primary Care Patients with Lower Respiratory Tract Infection: An Individual Patient Data Meta-Analysis
Source: PLoS One. 2016 Feb 26;11(2):e0149895. doi: 10.1371/journal.pone.0149895 (PMC4769284; doi:10.1371/journal.pone.0149895)
Supplement: S1 TRIPOD Checklist — Some of the items were not applicable (NA) to the current study. (PDF) [file pone.0149895.s001.pdf]

S1 Checklist. TRIPOD Checklist for prediction model development and validation with added text excerpts. Some of the items were not applicable (NA) to the current study.

Section/Topic	Item		Checklist Item	Page	Text Excerpt
**Title and abstract**					
Title	1	D;V	Identify the study as developing and/or validating a multivariable prediction model, the target population, and the outcome to be predicted.	1	"External Validation of Prediction Models for Pneumonia in Primary Care Patients with Lower Respiratory Tract Infection: An Individual Patient Data Meta-Analysis"
Abstract	2	D;V	Provide a summary of objectives, study design, setting, participants, sample size, predictors, outcome, statistical analysis, results, and conclusions.	4	OBJECTIVE/ STUDY DESIGN/ SETTING/ PARTICIPANTS/ OUTCOME: "In this study all published S&S models for prediction of pneumonia in primary care were externally validated in the individual patient data (IPD) of previously performed diagnostic studies."<br>SAMPLE SIZE: "N total=5308"<br>PREDICTORS: Not in abstract due to large number of predictors (page 9/10: "The prediction models included […] one model [Table 1 and S3 Table]").<br>STATISTICAL ANALYSIS: "Models were assessed […] average dataset performance ."<br>RESULTS: "Prediction models by […] lacked such correspondence."<br>CONCLUSIONS: "The model by […] for primary care use."
**Introduction**					
Background and objectives	3a	D;V	Explain the medical context (including whether diagnostic or prognostic) and rationale for developing or validating the multivariable prediction model, including references to existing models.	6/7	"Pneumonia is a major cause […]and recently developed models."
	3b	D;V	Specify the objectives, including whether the study describes the development or validation of the model or both.	7	"Therefore, a meta-analysis using individual patient data (IPD) from multiple studies was performed in order to extensively validate and compare the performance of all published S&S models for the diagnosis of pneumonia in primary care."
**Methods**					
Source of data	4a	D;V	Describe the study design or source of data (e.g., randomized trial, cohort, or registry data), separately for the development and validation data sets, if applicable.	7/8	"IPD for model validation […] the Cochrane Library (S1 Appendix)."
	4b	D;V	Specify the key study dates, including start of accrual; end of accrual; and, if applicable, end of follow-up.	7	"reference date: August 2012, 21st"
Participants	5a	D;V	Specify key elements of the study setting (e.g., primary care, secondary care, general population) including number and location of centres.	7/8	We included all "Prospective studies diagnosing pneumonia"
	5b	D;V	Describe eligibility criteria for participants.	7/8	"(a) were at least 18 years old; (b) presented trough self-referral in primary care, ambulatory care or at an emergency department with an acute or worsened cough (≤28 days of duration) or any other clinical presentation potentially caused by LRTI; (c) consulted for the first time for this disease episode; (d) were immunocompetent."
	5c	D;V	Give details of treatments received, if relevant.	NA	NA
Outcome	6a	D;V	Clearly define the outcome that is predicted by the prediction model, including how and when assessed.	8	"Eligible studies should have recorded clinical S&S and verified the disease status by CXR [21] or other imaging techniques."
	6b	D;V	Report any actions to blind assessment of the outcome to be predicted.	NA (19/S2)	NA for this meta-analytic approach. Blinding of outcome assessment of original development studies is described in discussion: "in most studies the CXR was review by a second blinded radiologist to minimize inter-observer variability [17,19,30,31,33,34]." See also S2 for quality assessment.
Predictors	7a	D;V	Clearly define all predictors used in developing or validating the	9/10	"The prediction models included […] one model [Table 1 and S3 Table]"

			multivariable prediction model, including how and when they were measured.		
	7b	D;V	Report any actions to blind assessment of predictors for the outcome and other predictors.	NA (S2)	Not described in detail. Is part of the QUADAS-2 quality assessment (Supporting Information S2)
Sample size	8	D;V	Explain how the study size was arrived at.	7/10/11	Method section: "IPD for model validation […] the Cochrane Library (S1 Appendix)." Result section: "Eighteen of the 3676 […] eight studies (N=5308) were included [17,19,30,31,33–36]."
Missing data	9	D;V	Describe how missing data were handled (e.g., complete-case analysis, single imputation, multiple imputation) with details of any imputation method.	8	"Missing values in IPD […] the models validated."
Statistical analysis methods	10a	D	Describe how predictors were handled in the analyses.	NA	NA
	10b	D	Specify type of model, all model-building procedures (including any predictor selection), and method for internal validation.	NA	NA
	10c	V	For validation, describe how the predictions were calculated.	8	"Pooled AUC was quantified […] AUC estimates [26]", ". The dAUC […] within an IPD dataset.", Calibration of included prediction models […] external validation process."
	10d	D;V	Specify all measures used to assess model performance and, if relevant, to compare multiple models.	8/9	The performance of included prediction […] in this group of patients."
	10e	V	Describe any model updating (e.g., recalibration) arising from the validation, if done.	NA	NA
Risk groups	11	D;V	Provide details on how risk groups were created, if done.	9	"Risk groups with a low (0-10%) predicted risk of pneumonia, an intermediate risk (10-30%) and a high risk (30-100%) were defined."
Development vs. validation	12	V	For validation, identify any differences from the development data in setting, eligibility criteria, outcome, and predictors.	12	Difference in setting, outcome and predictors are described in Table 2.
**Results**					
Participants	13a	D;V	Describe the flow of participants through the study, including the number of participants with and without the outcome and, if applicable, a summary of the follow-up time. A diagram may be helpful.	11/Fig 1/Table 1	Overview of selection of IPD: Figure 1<br>Participants: "The study by van Vugt et al. contained 55% […] ranged from 5% to 43%", Table 2
	13b	D;V	Describe the characteristics of the participants (basic demographics, clinical features, available predictors), including the number of participants with missing data for predictors and outcome.	11	"Of the eight included studies […] the outcome pneumonia."
	13c	V	For validation, show a comparison with the development data of the distribution of important variables (demographics, predictors and outcome).	Table 1	Table 1 shows the comparison of most of the characteristics the development data and the IPD used. No formal comparison of characteristics is made due to number of studies and models included.
Model development	14a	D	Specify the number of participants and outcome events in each analysis.	NA	NA
	14b	D	If done, report the unadjusted association between each candidate predictor and outcome.	NA	NA
Model specification	15a	D	Present the full prediction model to allow predictions for individuals (i.e., all regression coefficients, and model intercept or baseline survival at a given time point).	NA	NA
	15b	D	Explain how to the use the prediction model.	NA	NA
Model performance	16	D;V	Report performance measures (with CIs) for the prediction model.	13/14	Reported
Model-updating	17	V	If done, report the results from any model updating (i.e., model specification, model performance).	NA	NA
**Discussion**					
Limitations	18	D;V	Discuss any limitations of the study (such as nonrepresentative sample, few events per predictor, missing data).	20	"A potential limitation […] with the primary aim of our study."

Interpretation	19a	V	For validation, discuss the results with reference to performance in the development data, and any other validation data.	17/18/Table 3	“It is common that performance […] be validated in these dataset”
	19b	D;V	Give an overall interpretation of the results, considering objectives, limitations, results from similar studies, and other relevant evidence.	17	“It is common that performance […]second blinded radiologist to minimize inter-observer variability [17,19,30,31,33,34].”
Implications	20	D;V	Discuss the potential clinical use of the model and implications for future research.	16	“Prediction models can […] use in primary care.”
**Other information**					
Supplementary information	21	D;V	Provide information about the availability of supplementary resources, such as study protocol, Web calculator, and data sets.	Supplements S1-S5	NA
Funding	22	D;V	Give the source of funding and the role of the funders for the present study.	25	“No direct funding […] manuscript.”

*Items relevant only to the development of a prediction model are denoted by D, items relating solely to a validation of a prediction model are denoted by V, and items relating to both are denoted D;V. We recommend using the TRIPOD Checklist in conjunction with the TRIPOD Explanation and Elaboration document.
