# Supplementary material for: External Validation of Prediction Models for Pneumonia in Primary Care Patients with Lower Respiratory Tract Infection: An Individual Patient Data Meta-Analysis
Source: PLoS One. 2016 Feb 26;11(2):e0149895. doi: 10.1371/journal.pone.0149895 (PMC4769284; doi:10.1371/journal.pone.0149895)
Supplement: S1 Appendix — (PDF) [file pone.0149895.s002.pdf]

S1 Appendix. Search strategies for PubMed, EMBASE and the Cochrane Library.

General:

Reference date: 21-08-2012

No language or preset filters used

Filters in Pubmed and EMBASE: Haynes et al. and Wilcyinski et al. [21,22].

Mesh term were first mapped and subsequently added to syntax.

PubMed:

(sensitiv*[Title/Abstract] OR sensitivity and specificity[MeSH Terms] OR diagnos*[Title/Abstract] OR diagnosis[MeSH:noexp] OR diagnostic *[MeSH:noexp] OR diagnosis, differential[MeSH:noexp] OR diagnosis[Subheading:noexp]) AND (c-reactive[tiab] OR reactive protein[tiab] OR CRP[tiab] OR C-Reactive Protein[Mesh]) AND (Pneumonie[tiab] OR Pneumonia[tiab] OR Pneumoniae[tiab] OR Pneumonitis[tiab] OR Pneumonias[tiab] OR Airway infection[tiab] OR Airway infections[tiab] OR Airway inflammation[tiab] OR Airway inflammations[tiab] OR Lower respiratory tract infection[tiab] OR Lower respiratory tract infections[tiab] OR Lower respiratory infection[tiab] OR Lower respiratory infections[tiab] OR Lower airway infection[tiab] OR Lower airway infections[tiab] OR Lower airway inflammation[tiab] OR Respiratory infection[tiab] OR Respiratory infections[tiab] OR Respiratory inflammation[tiab] OR Respiratory inflammations[tiab] OR Respiratory tract infection[tiab] OR Respiratory tract infections[tiab] OR Respiratory tract inflammation[tiab] OR Respiratory tract inflammations[tiab] OR LRTI[tiab] OR LRTIS[tiab] OR RTI[tiab] OR RTIS[tiab] OR respiratory tract illness[tiab] OR Pneumonia[Mesh] OR Respiratory Tract Infections[Mesh])

EMBASE:

('pneumonie':ab,ti OR 'pneumonia':ab,ti OR 'pneumoniae':ab,ti OR 'pneumonitis':ab,ti OR 'pneumonias':ab,ti OR 'airway infection':ab,ti OR 'airway infections':ab,ti OR 'airway inflammation':ab,ti OR 'airway inflammations':ab,ti OR 'lower respiratory tract infection':ab,ti OR 'lower respiratory tract infections':ab,ti OR 'lower respiratory infection':ab,ti OR 'lower respiratory infections':ab,ti OR 'lower airway infection':ab,ti OR 'lower airway infections':ab,ti OR 'lower airway inflammation':ab,ti OR 'respiratory infection':ab,ti OR 'respiratory infections':ab,ti OR 'respiratory inflammation':ab,ti OR 'respiratory inflammations':ab,ti OR 'respiratory tract infection':ab,ti OR 'respiratory tract infections':ab,ti OR 'respiratory tract inflammation':ab,ti OR 'respiratory tract inflammations':ab,ti OR 'lrti':ab,ti OR 'lrtis':ab,ti OR 'rti':ab,ti OR 'rtis':ab,ti OR 'respiratory tract illness':ab,ti OR 'pneumonia'/exp OR 'respiratory tract infections'/exp) AND ('specificity' OR predict* OR 'diagnosis':lnk) AND ('c-reactive':ab,ti OR 'reactive protein':ab,ti OR 'crp':ab,ti OR 'c-reactive protein'/exp)

Cochrane:

#1:(pneumonie:ab,ti) OR (pneumonia:ab,ti) OR (pneumoniae:ab,ti) OR (pneumonitis:ab,ti) OR (pneumonias:ab,ti) OR (airway infection:ab,ti) OR (airway infections:ab,ti) OR (airway inflammation:ab,ti) OR (airway inflammations:ab,ti) OR (lower respiratory tract infection:ab,ti) OR (lower respiratory tract infections:ab,ti) OR (lower respiratory infection:ab,ti) OR (lower respiratory infections:ab,ti) OR (lower airway infection:ab,ti) OR (lower airway infections:ab,ti) OR (lower airway inflammation:ab,ti) OR (respiratory infection:ab,ti) OR (respiratory infections:ab,ti) OR (respiratory inflammation:ab,ti) OR (respiratory inflammations:ab,ti) OR (respiratory tract infection:ab,ti) OR (respiratory tract infections:ab,ti) OR (respiratory tract inflammation:ab,ti) OR (respiratory tract inflammations:ab,ti) OR (lrti:ab,ti) OR (lrtis:ab,ti) OR (rti:ab,ti) OR (rtis:ab,ti) OR (respiratory tract illness:ab,ti)

#2: (pneumonia[Mesh])

#3: (respiratory tract infections[Mesh]))

#4: (C-Reactive Protein[Mesh])

#5: (c-reactive:ab,ti) OR (reactive protein:ab,ti) OR (CRP:ab,ti) OR

Final syntax cochrane: (#1 OR #2 OR #3) AND (#4 OR #5)
