# Supplementary material for: External Validation of Prediction Models for Pneumonia in Primary Care Patients with Lower Respiratory Tract Infection: An Individual Patient Data Meta-Analysis
Source: PLoS One. 2016 Feb 26;11(2):e0149895. doi: 10.1371/journal.pone.0149895 (PMC4769284; doi:10.1371/journal.pone.0149895)
Supplement: S1 Protocol — (PDF) [file pone.0149895.s003.pdf]

DIAGNOSTIC VALUE OF C-REACTIVE PROTEIN FOR PNEUMONIA IN PRIMARY CARE ACUTE COUGH PATIENTS: AN INDIVIDUAL PATIENT DATA META-ANALYSIS

*

STUDY PROTOCOL

Authors:
Alwin Schierenberg, Margaretha C Minnaard, MD, Alma C van de Pol, MD, PhD, Niek J de Wit, MD, PhD, Joris AH de Groot, PhD, Theo JM Verheij, MD, PhD

Affiliation:
Julius Center for Health Sciences and Primary Care, UMC Utrecht, Utrecht, the Netherlands

Date: October 2012
Version: draft v2.6

Abstract

Background. In primary care, lower respiratory tract infections (LRTIs) are the principal reason for consultation, however there is little consensus on the optimal diagnostic strategy for pneumonia. This can lead to the delayed start of treatment or to improper prescription of antibiotics.

Objectives. Our main objective is to quantify the value of C-reactive protein (CRP) for the diagnosis of pneumonia in adults presenting with acute cough in primary care by determining the 1) accuracy of existing pneumonia prediction models based on signs and symptoms alone?, 2) single test accuracy of CRP overall and in clinical relevant subgroups 3) added value of CRP beyond signs and symptoms by performing an individual patient data meta-analysis (IPDMA) of all available data.

Search methods. Electronic databases PUBMED, EMBASE and the Cochrane Library were searched free text or MeSH for "CRP" and "pneumonia" or "LTRI", with the use of a diagnostic filter.

Selection criteria. Studies will be included when a) concerning adult patients directly presenting in a primary care setting, ambulatory care setting or at an emergency department with acute cough disease or suspected of LRTI b) reporting the type of disease diagnosed, c) reporting on the cross-sectional relationship between CRP and the presence or absence of pneumonia, d) reporting or presence of individual data on clinically relevant history, symptoms and signs, e) the authors are willing and able to provide their individual patient data. Patients will be excluded when diagnosed with co morbidities known to influence CRP or LRTI pathoetiology.

Data collection and analysis. Two individual investigators performed inclusion of studies by abstract screening and full text reading, as well as quality assessment according the QUADAS-2 scoring systems. Statistical analysis will include 1) calibration and discrimination of existing pneumonia prediction models, 2) Single test accuracy a univariable analysis of symptoms, signs and CRP, determining the sensitivity, specificity, PPV, NPV and optimal threshold for continuous variables (e.g. CRP), 3) determination of difference in accuracy between subgroups of patients, 4) selection of determinants for multivariable analysis on the bases of clinical relevance, ease of measurement and AIC, 5) a multivariable analysis with determinants from history, symptoms and signs, 6) calibration and discrimination performance of a reduced model, 7) determination of the added value of CRP in combination with the reduced model, 8) internal validation of the extended model using bootstrapping, 9) NRI, IDI and c-statistic to indicate reclassification.

Search results. Of the 3683 articles identified after searching electronic databases, 3546 studies were excluded after screening the titles, 85 studies were excluded after abstract reading. Twelve of the remaining 52 studies were eligible for inclusion. Seven articles were identified via additional sources, of which one was included. A total of 13 (preliminary) articles were included in the qualitative synthesis.

Discussion. Combing individual patients data from different observational studies will provide additional valuable and clinically useful information on the diagnostic value of CRP in patients presenting with LRTIs in primary care and in relevant subgroups.

Background

Target condition

In adults patients with acute cough presenting in primary care, the diagnosis of pneumonia is important because it is the major infection-related cause of death in developed countries (1, 2) and calls for specific treatment, while in patients with, e.g. acute bronchitis, the disease is self-limiting (3). With an incidence of 34 per 1000 patients per year (4), LRTIs are the principal reason for consultation of a General Practitioner (GP) (5). This illustrates the demand on treatment capacity in daily practice, as well as the need for adequate diagnostic tools. While it is not feasible to perform chest radiographs (CXR) in all patients suspected of a LRTI, other tests are being suggested and investigated for this purpose. For example a novel Point-of-Care Test (POCT) has been proposed to adequately differentiate between pneumonia and other LRTIs (6-8). This POCT is based on the widely used inflammation parameter C-Reactive Protein (CRP).

Index test

CRP is an acute phase protein synthesized by the liver in response to inflammation within 6-8 hours (9). CRP measurement is increasingly used in primary care to assist GPs in decision-making in various infection domains (6, 10). A POCT enables the general practitioner to measure CRP levels within 2 minutes (11). CRP plays a central role in the in 2011 initiated CaTCH project, which aims to evaluate the implementation of such a CRP POCT in primary care by investigating the validity of different POCT devices, the diagnostic value of CRP in adults and children and the effects of implementation on antibiotic prescription; what patients groups are influenced and what policy is initiated following the test.

Alternative test(s)

In adults procalcitonin is not superior to CRP in identifying pneumonia, bacterial etiology, or adverse outcome (12)(van Vugt et al. 2012, under review). In comparison to other laboratory tests like ESR and leukocyte count, CRP has superior discrimination properties for bacterial infection (13-15). Therefore, it is suggested that CRP will be of greater help in discrimination between pneumonia and other LTRIs.

Rationale

There are several reasons why the diagnostic value of CRP for pneumonia in primary care should be studied more extensively (Box 1). First, current diagnosis of pneumonia depends on the clinical presentation of the patient. However, a systematic review indicated a lack of sensitivity of these clinical signs and symptoms for an accurate diagnosis of pneumonia (16). Even a combination of criteria resulted in a sensitivity of less than 50 percent when using chest X-ray (CXR) as the reference

test Also, improper prescription of antibiotics leads to increased costs, undesired side effects of medication, more patients expecting antibiotic treatment in a subsequent episode and increased risk of antibiotic resistance of bacteria (17, 18). This stresses the need for an additional test that can be performed without being demanding for the patient, with low costs and adequate measurement of CRP. A CRP POCT seems to suffice on these points. Falk *et al.* conclude in their systemic review that "the evidence for the benefits of POC CRP measurement in LRTI patients in primary care is limited, contradictory and does not support its use to guide treatment yet" (19). However, this review and three additional recent reviews (11, 19-21) did not include more recently published studies (22) (van Vugt et al. 2012, under review). Moreover, based on the current literature pneumonia seems unlikely when CRP levels are below 20 mg/l in a diagnostic signs and symptoms model (19). However, due to the diversity of cut-off values used in the literature (11, 21), it remains uncertain what policy should be pursued when CRP values are between 20-100 mg/l. Therefore, determination of optimal cut-off values is warranted.

Furthermore, several factors can influence CRP levels, defining different subgroups of patients. For example, increased age (23), asthma (24) and COPD(25) are associated with increased CRP as well as increased incidence of CAP (26, 27). The identification of appropriate cut-off values and subgroup analysis are difficult or impossible to address in a meta-analysis of aggregate data, but can be addressed in a meta-analysis of individual patient data. This makes it the appropriate type of study for the investigation of our research questions.

Individual patient data meta-analyses have been increasingly published in recent decades, now averaging 49 articles a year (28) and widely considered to be the gold standard for systemic reviews (29, 30), generating the highest quality of evidence. Additional advantages to an aggregate meta-analyses in this context are a) examination of the added value of CRP in existing models (validation) and in novel multivariable models, b) improved assessment of study quality and study design, leading to better understanding of validity of result across patients groups, c) improved exploration of heterogeneity on patient level d) the identification and use of unpublished data, e) consistent variable selection across studies (31, 32). Therefore, we propose to perform a meta-analysis of individual patient data to investigate the value of CRP in acute cough primary care patients.

**Box 1. Summary of rationale**
1. Need for additional tests to diagnose CAP in primary care<br>2. No consensus of diagnostic and added value of CRP to diagnose pneumonia in primary care<br>3. An IPD meta-analysis provides specific advantages over an aggregate meta-analysis due to:<br>➢ Identification and use of unpublished data<br>➢ Better assessment of study quality and study design, which leads to better understanding of validity of result across patients groups<br>➢ Improved investigation of differences in accuracy between subgroups of patients

- Better identification of optimal cut-off points
- Consistent variable selection across studies to examine multivariable models
- Added value of CRP in existing models and in newly developed multivariable models
- More powered subgroup analysis

Objectives

To answer our research question: ”what is the diagnostic value for pneumonia of CRP in primary care?”, we have postulated several objectives. First, we will determine the diagnostic value of existing “signs and symptoms” models in the IPD population. Secondly, we will determine the single test accuracy of all available variables and set the optimal threshold for CRP. Thirdly, determine the added value of CRP to relevant determinants.

Methods

Criteria for considering studies for this review

Study types

Studies examining the cross-sectional relationship between CRP levels and the presence or absence of LRTI are included. Studies using the separate inclusion of healthy controls or studies with less than 10 participants are excluded. When duplicate publications are identified, the study with the highest number of participants will be included. No language restriction is used.

Only studies that are conducted in healthcare settings that provide care for non-referred patients are eligible for inclusion. These include general practitioner offices, emergency departments, ambulatory care and other private practices. These types of healthcare settings are chosen because of increased generalization of the study results across different countries (i.e. different systems of primary healthcare), while maintaining adequate focus on the intended study population (i.e. patients with acute cough in primary care). It is pursued to include study data that describe a pre-defined minimum of symptoms, signs and comorbidities. When studies are lacking variables, datasets are considered to be incomplete, meaning they can be excluded for specific statistical analysis involving this variable. If appropriate and possible, partly missing variables on study level, will be imputed.

Participants

Patients eligible for inclusion (see Box 2) in statistical analysis are a) at least 18 years old (adults) and present with b) an acute or worsened cough (≤28 days of duration) as primary symptom, or any other clinical presentation considered by the first consulted health-care provider in general practice, ambulatory care or at an emergency department to be caused by LRTI and c) consulting for the first time for this illness episode (i.e. non-referred patients). Studies are excluded when more than 5% of

the subjects have an active pregnancy, immunodeficiency disorder, antibiotic prophylaxis, immune suppressive therapy, severe illness (MI, unstable AP, malignancy), hospital acquired pneumonia, been treated in ICU, systemic inflammatory disorders (e.g. RA, SLE, polymyositis, primary Sjögren's syndrome, acute leukemia, and ulcerative colitis or liver failure). If their datasets will allow exclusion of these particular subjects the remaining subjects are included for this review.

Index tests

The index test used in this review is C-reactive Protein. In general laboratory analyzers measure CRP. In a primary care setting CRP is measures mainly by POCT systems. Both types of measurement are considered suitable for use in general practice (Minnaard et al. 2012, under review) and therefore both included in this review. No comparator tests are included in this study.

Target conditions and reference standards

The target condition is community-acquired pneumonia (CAP), which is typically defined as an infection of the alveolar or gas-exchanging portions of the lungs occurring outside the hospital, with clinical symptoms accompanied by the presence of an infiltrate in the chest radiograph (L.A. Mandell, R.G. Wunderink, A. Anzueto, J.G. Bartlett, G.D. Campbell, N.C. Dean et al Infectious Diseases Society of America; American Thoracic Society. Infectious Diseases Society of America/American Thoracic Society consensus guidelines on the management of community-acquired pneumonia in adults Clin Infect Dis, 44 (2007), pp. S27–S72). Chest radiograph (CXR) here is regarded as the reference test for the diagnosis of pneumonia in outpatients, and regarded as common practice for a pneumonia suspicion (33). Other diagnostic tools are allowed if deemed adequate for the diagnosis of pneumonia. These types of alternative reference standards will be discussed where appropriate. Studies using computer tomography (CT) or magnetic resonance imaging (MRI) as reference standard are also included. Etiologic laboratory test will not be considered as reference test, as they frequently tend to test falsely negative and are often nonspecific (33). Moreover, these tests are not of main interest of the current study domain and setting. No minimum or maximum of days between first consultation and diagnosis by the reference standard is set. Diagnosis of either lobar or bronchopneumonia will be indicated as 'pneumonia', while other LRTIs will be indicated as 'pneumonia absent'.

**Box 2. Inclusion and exclusion criteria**
**Inclusion criteria**<br>• Patient is at least 18 years old<br>• Patient has an acute or worsened cough (≤28 days of duration) as primary symptom, or any other clinical presentation considered by the first consulted health-care provider to be caused by LRTI<br>• Patient is consulting for the first time for this illness episode (non-referred patients)<br>• Patient presents in a primary care setting, ED, or any other setting accepting non-referred patients

- Observational study design (N≥10)
- CRP is measured
- A reference test is used (either CXR, CT, MRI, or any other suitable test)

Exclusion criteria

- More than 5% of the subjects have one of the following conditions and cannot be identified and removed from the dataset:
    - An active pregnancy
    - Immunodeficiency disorder
    - Antibiotic prophylaxis
    - Immune suppressive therapy
    - Severe illness (MI, unstable AP, malignancy)
    - Treatment in ICU
    - Systemic inflammatory disorders (e.g. RA, SLE, polymyositis, primary Sjögren's syndrome, acute leukemia, and ulcerative colitis or liver failure)

Search methods for identification of studies

Electronic searches

We searched MEDLINE, EMBASE and the Cochrane Library using an electronic search that incorporates indexing terms and plain text words for the index test ("CRP") and target condition ("pneumonia"). Terms describing "LRTI" were added to the target condition terms while articles may report different outcomes (e.g. antibiotics prescription) but do use a CRP test to diagnose patients presenting with LRTIs. A filter was implemented to identify diagnostic studies (34, 35). The search strategy was developed in collaboration with a medical information specialist (BK) and was refined until all references in previously identified systemic reviews of CRP for CAP (11, 19-21) were listed and no additional cross-references via full-text reading of newly identified reviews were found. No additional resources will be used to identify unpublished studies, however, contributing authors are encouraged to inform about missing studies. The final search strategies for these databases are presented in Appendix 1.

Data collection and analysis

Selection of studies

Retrieved studies were independently screened for relevance by AS through applying the selection criteria to the title. Eligible studies were screened on abstract and than selected studies were read in full-text by two reviewers independently (AS, JdG). Discrepancies were resolved by discussion or, if necessary, evaluated by a third reviewer (ThV). In the final article a PRISMA flow chart will be included, illustrating the study selection process (Appendix 2).

Data acquisition and management

The first author and a secondary author of choice (max. of two) of all relevant papers will be invited to take part in the IPD, when the respective complete dataset is available by September 31st 2012. If the authors incline to participate, a detailed study protocol is provided and they are asked for their complete datasets, minimizing their efforts to engage in dataset formatting and optimizing the amount of available information. All types of dataset formats are accepted. Variables of the complete dataset should be adequately labeled within the dataset or in a separate data dictionary. Also a list of provisionally included studies is provided and authors will be asked to identify missing, unpublished or studies that are in progress. To safeguard patient privacy cooperating authors are requested to remove patient names and contact details and be replaced by unique ID numbers before data is supplied.

If already identified authors or additional authors are unwilling to cooperate or do not respond, differences are examined between studies that provided individual patient data and studies that did not by using meta-analysis methods that combine IPD and aggregate data (36, 37).

Patient origin will be visualized in the STARD flow-chart (Appendix 3). Key characteristics of the selected studies will be summarized in a table. The following characteristics will be considered: age and gender distribution, pre-test prevalence of pneumonia, type of CRP test, diagnostic criteria LTRI, reference standard, place of initial consultation.

Assessment of methodological quality

In order to appropriately assess study quality the research protocols of collaborating authors are requested. Subsequently, we will determine the quality of the original studies using the QUADAS-2 tool (Appendix 4). Two reviewers (AS and JdG) will independently apply the QUADAS-2 tool. Discrepancies will be solved by discussion or, is necessary, evaluated by a third reviewer (ThV). In case of unclear or undisclosed information in regard to study quality the authors will be contacted. The outcomes of the QUADAS-2 tool are depicted for all four domains and scored either with low, high or unclear risk of bias (Appendix 4). When database compilation is completed we will perform data checks on single variables, simple tables and plots in order to assess the reproducibility of the reported accuracy in the study. When values are missing or invalid, authors are first contacted for clarification. Unresolved missing values will be evaluated and values missing at random (MAR) or missing completely at random will be imputed using multiple imputation methods. Variables missing at study level will be estimated when appropriate (Currently investigated by Sanne Peters and Thomas Debray).

Statistical analysis and data synthesis

1. *Determine the diagnostic value of existing "signs and symptoms" models in the IPD population.*

Five different models were previously identified by the GRACE study (van Vugt et al. 2012, under review) to be fit for use in primary care (Diehr et al., 1984; Heckerling et al., 1990; Hopstaken et al., 2003; Melbye et al., 1992; Singal et al., 1989). Each of these models will be externally valdiated using our IPD database. In our validation we will examine discrimination and calibration of the identified model. Calibration will be assessed by examining the agreement in predicted probabilities by the model and the observed probabilities in each of the valdation datasets and by fitting the original risk score as a single predictor in the valdiation datasets. Discrimination will be assessed by calculating the AUC of the original risk score when it is used as a single predictor in the valdiation datasets and compared with the AUC of the original model.

With these models the diagnostic probability for pneumonia will be calculated for all inlcuded patients (N=..), and the area under the receiver operating characteristic curve (AUC) with 95% confidence intervals (95% CI) will be calculated. All models will be assesed for their goodness-of-fit using Hosmer and Lemeshow (HL) statistic and by examination of the calibration plot, because of possible lack of statistical power of HL statistic.

2. *Develop a novel "signs and symptoms" model for the diagnosis of CAP in primary care.*

Univariable analysis

It is expected that existing models will perform sub-optimally (38). Therefore, a selection of variables will be made that make a contribution to the discrimination between pneumonia presence and absence. Previously identified variables are: age, gender, smoking, cough, severe cough, phlegm, breathlessness, runny nose absent, fever, chest pain, diarrhoea, interference with daily activities, any comorbidity, general toxicity, diminished vesicular breathing, crackles, tachycardia, tachypnoea, low blood pressure (<90/60), temperature (>37.8 C). Using these diagnostic variables univariable odds ratios (ORs) with 95% CIs will be calculated for each candidate predictor, using logistic regression modelling. For continuous variables restricted cubic splines will be used to determine the relationship with outcome when continuous variables are, for example, not linear or follow a clear log distribution. (39). Optimal cut-off values for CRP will be determined by using the Youden Index and will be presented together with their sensitivity, specificity, PPV, NPV. To correct for non-random differences between studies multilevel logistic regression techniques will be used. These results will be compared to a standard multivariable regression analysis, and if no differences arise the latter approach will be used. A random-effect model will be used in all calculations.

Multivariable analysis

All preselected diagnostic variables will be entered in a multilevel or multivariable logistic regression analysis. A selection will be made on the bases of clinical relevance, ease of measurement and by

using the Akaike Information Criterion (AIC), a reduced model will be fitted and the AUC and goodness-of-fit will be computed.

3. *Determine the added value of CRP in the diagnosis of CAP in primary care.*

Using the reduced model together with CRP, the added value of CRP at the optimal thresholds, will be calculated, which will be indicated by a multivariable ORs and AUCs.
The number of correctly reclassified patients after the addition of CRP to the model will be expressed in the net reclassification improvement (NRI), NRI, IDI, c-statistic

It will be investigated if a Classification and Regression Tree (CART) providing an easy-to-use decision rule for primary care physicians is feasible and reflects a realistic approach of the diagnostic value of the studies determinants.

Box 3.Hierarchical Plan per Objective.

1. *Determine the diagnostic value of existing "signs and symptoms" models in the IPD population.*
    a. Calibration ,Goodness of fit (HL test) and examination of the calibration plot (due to lack of statistical power of HL test)
    b. Discrimination, ROCs/AUCs
2. *Develop a novel diagnostic prediction model for the diagnosis of CAP in primary care.*
    a. Univariable analysis
        i. Determine thresholds of CRP (Youden Index) or categorical cut-off value.
        ii. Sensitivity, specificity, PPV, NPV of CRP at a several fixed cut-off value across studies with pooled estimates
        iii. AUC's of logistic models only including CRP for each study + random effects pooled estimate
        iv. Individual and pooled ROC curve of CRP. To obtain a summary ROC-curve based on all available data and to assess whether specific covariates have an impact on the diagnostic accuracy, receiver operating characteristics (ROC) regression analysis will be performed. The hierarchical nature of the data (i.e data from different studies) was preserved by first comparing CRP between patients with (cases) and without the target condition (controls) within each study. CRP levels in control groups were then used to standardize the biomarker levels in cases within each corresponding study. These standardized values of cases and controls were then analyzed using logistic regression as described by Jones and Pepe (Janes H, Pepe MS: Adjusting for covariates in studies of diagnostic, screening, or prognostic markers: An old concept in a new setting. Am J Epidemiol 168:89-97, 2008)
        v. Difference in accuracy between subgroups of patients
            a. Increase in AUC within studies and pooled across studies
    b. Multivariable analysis

i. Select determinants for multivariable analysis with one or several methods:

1. Methods:

a. $P<0.05/0.20/0,25$ (Backward selection)

b. On the bases of clinical relevance and ease of measurement.

c. Using the Akaike Information Criterion (AIC)

d. Guided combination (Harrell 2001, Steyerberg 2004)

2. Use shrinkage before or after multivariable analysis.

a. Multivariable analysis could be done with many variables as population is large (N~4000+), shrinkage is optional

ii. Determinants: add in logic order; history>symptoms>signs (>tests)

iii. Reduce model

1. Exclude with log likelihood ratio test, at liberal level (self-set p level)

2. Estimate diagnostic accuracy of reduced model

iv. Internal validation extended model

1. Bootstrapping

2. Shrinkage factor (i.e. more conservative model)

v. Discrimination, AUCs

vi. Calibration , Goodness of fit (HL test) and examination of the calibration plot (due to lack of statistical power of HL test)

vii.

3. *Determine the added value of CRP in the diagnosis of CAP in primary care*

a. Extended = (final) diagnostic prediction model +CRP

i. Sensitivity, specificity, PPV, NPV of extended model

b. Difference in accuracy between subgroups of patients

i. Increase in AUC within studies and pooled across studies

c. Reclassification

i. NRI, IDI, c-statistic

Investigations of heterogeneity

We will explore several study characteristics as potential sources of heterogeneity, including: “high” *versus* “low” quality studies, inclusion criteria for LTRI/acute cough, methods of diagnosis of CAP, correlation with age (sig. different between studies), inclusion/exclusion differences of patients between studies and other relevant sources. The type of analyzer used will not be investigated as a source of heterogeneity, whereas a recent study indicated fractional differences in test results (Minnaard et al. 2012, under review).

Subgroup analysis

To our knowledge no research has been focusing on relevant subgroups in LRTI patients. Electronic databases were explored to identify related research. Several subgroups are being suggested for further

analysis: asthma, COPD, smoking, different age categories, outcome and treatment related subgroups (e.g. ABx treatment), viral vs. bacterial pathogens, other subgroups identified in literature.

Publication policy

The coordinating centre is responsible for the collection, maintenance and pooling of the data provided by collaborating investigators, for leading and conducting the statistical analyses and writing of manuscripts for publications. The coordinating centre only has direct access to the combined dataset.
The coordinating centre will send reports (including recent developments, interim analyses and results) by email to the participating investigators on a regular basis. Participating investigators have the opportunity to comment on these reports and to provide extra input to the coordinating centre.
Manuscripts resulting from the collaboration will be sent to all participating investigators prior to submission for publication, for comments and agreement. In case disagreement, the following will be applied; the report should present the results of the IPD meta-analysis, outlining all available evidence. Only interpretations of the data that are unanimously agreed on by all collaborators will be included. Any collaborating group is free to withdraw their data from the IPD meta-analysis at all times.
Any publications arising from the IPD meta-analysis are in the name of all participating investigators. The principal investigator from each of the collaborating studies will be a co-author of the current study. If the number of authors is conflict with the criteria of the journal chosen for submission, a collaborative group of authors will be formed. Furthermore, acknowledgements will be made to other investigators from the collaborating studies that made important contributions to the original studies. A summary of practical issues can be found in Box 4.

Results

Results of the search

Of the 3683 articles identified after searching electronic databases, 3546 studies were excluded after screening the titles, 85 studies were excluded after abstract reading. Twelve of the remaining 52 studies were eligible for inclusion. Seven articles were identified via additional sources, of which one was included. A total of 13 articles were eligible for inclusion after full text reading.

Discussion

The proposed IPD meta-analysis is necessary to determine the diagnostic accuracy of CRP for pneumonia in primary care acute cough patients. Combing individual patients data from different observational studies will provide additional valuable and clinically useful information and possibly identify relevant patient subgroups. We currently aim to include 5000 patients in the analysis and anticipate this will provide definitive data synthesis, which will enable clinical practice and future research.

Acknowledgements

BK – Bianca Kramer, information specialist, commented and developed the search strategy for the systemic search

Conflict of interest

No conflict of interest is currently reported by any of the authors.

**Box 3. Practical issues for IPD meta-analysis participation**
❒ I have read the study protocol and do agree with its content or have notified the authors with my remarks
❒ Data library containing variable coding structure enclosed
❒ Study protocol submitted to coordination investigator.
❒ Dataset check for latest version
❒ Information on non-published data or follow-up data enclosed
❒ Consent form signed and send back
**Security issues:**
❒ Patient name changed by unique ID
❒ All files converted into 1 encrypted .ZIP file (recommended WinZIP[a])
❒ Password E-mailed to secondary E-mail account (address: ….)
[a] Can be downloaded from <www.winzip.com/downwz.htm>

References

1. Niederman MS, Mandell LA, Anzueto A, Bass JB, Broughton WA, Campbell GD, et al. Guidelines for the management of adults with community-acquired pneumonia. Diagnosis, assessment of severity, antimicrobial therapy, and prevention. American journal of respiratory and critical care medicine. 2001;163(7):1730-54. Epub 2001/06/13.

2. Mortensen EM, Coley CM, Singer DE, Marrie TJ, Obrosky DS, Kapoor WN, et al. Causes of death for patients with community-acquired pneumonia: results from the Pneumonia Patient Outcomes Research Team cohort study. Archives of internal medicine. 2002;162(9):1059-64. Epub 2002/05/09.

3. Smucny J, Fahey T, Becker L, Glazier R. Antibiotics for acute bronchitis. Cochrane Database Syst Rev. 2004(4):CD000245. Epub 2004/10/21.

4. Van der Linden MW WG, De Bakker DH, Schellevis FG. Tweede Nationale Studie naar ziekten en verrichtingen in de huisartspraktijk: klachten en aandoeningen in de bevolking en in de huisartspraktijk. Utrecht/Bilthoven: NIVEL/RIVM, 2004.

5. Macfarlane JT, Holmes WF, Macfarlane RM. Reducing reconsultations for acute lower respiratory tract illness with an information leaflet: a randomized controlled study of patients in primary care. Br J Gen Pract. 1997;47(424):719-22. Epub 1998/03/31.

6. Verheij ThJM HR, Prins JM, Salomé PhL, Bindels PJ, Ponsioen BP †, Sachs APE, Thiadens HA, Verlee E. NHG-Standaard Acuut hoesten (Eerste herziening). Huisarts Wet: 2011 Contract No.: 68.

7. Hopstaken RM, Muris JW, Knottnerus JA, Kester AD, Rinkens PE, Dinant GJ. Contributions of symptoms, signs, erythrocyte sedimentation rate, and C-reactive protein to a diagnosis of pneumonia in acute lower respiratory tract infection. The British journal of general practice : the journal of the Royal College of General Practitioners. 2003;53(490):358-64.

8. Flanders SA, Stein J, Shochat G, Sellers K, Holland M, Maselli J, et al. Performance of a bedside c-reactive protein test in the diagnosis of community-acquired pneumonia in adults with acute cough. The American Journal of Medicine. 2004;116(8):529-35.

9. Clyne B, Olshaker JS. The C-reactive protein. Journal of Emergency Medicine. 1999;17(6):1019-25.

10. Berger MY, De Wit NJ, Vogelenzang R, Wetzels RV, Van Rijn-van Kortenhof NMM, Opstelten W. The NHG Guideline diverticulitis. Huisarts Wet. 2011(2011:54(9):):492-9.

11. Engel MF, Paling FP, Hoepelman AIM, van der Meer V, Oosterheert JJ. Evaluating the evidence for the implementation of C-reactive protein measurement in adult patients with suspected lower respiratory tract infection in primary care: a systematic review. Family practice. 2011.

12. Holm A, Pedersen SS, Nexoe J, Obel N, Nielsen LP, Koldkjaer O, et al. Procalcitonin versus C-reactive protein for predicting pneumonia in adults with lower respiratory tract infection in primary care. Br J Gen Pract. 2007;57(540):555-60. Epub 2007/08/31.

13. Hellgren U, Julander I. Are white blood cell count, platelet count, erythrocyte sedimentation rate and C-reactive protein useful in the diagnosis of septicaemia and endocarditis? Scandinavian Journal of Infectious Diseases. 1986;18(5):487-8.

14. Hogevik H, Olaison L, Andersson R, Alestig K. C-reactive protein is more sensitive than erythrocyte sedimentation rate for diagnosis of infective endocarditis. Infection. 1997;25(2):82-5.

15. Korppi M, Heiskanen-Kosma T, Leinonen M. White blood cells, C-reactive protein and erythrocyte sedimentation rate in pneumococcal pneumonia in children. The European respiratory journal : official journal of the European Society for Clinical Respiratory Physiology. 1997;10(5):1125-9.

16. Metlay JP, Fine MJ. Testing strategies in the initial management of patients with community-acquired pneumonia. Annals of internal medicine. 2003;138(2):109-18. Epub 2003/01/17.

17. Bradley JS, Guidos R, Baragona S, Bartlett JG, Rubinstein E, Zhanel GG, et al. Anti-infective research and development--problems, challenges, and solutions. The Lancet infectious diseases. 2007;7(1):68-78. Epub 2006/12/22.

18. Tacconelli E, De Angelis G. Fighting antibiotic resistance all over Europe. Expert review of anti-infective therapy. 2010;8(7):761-3. Epub 2010/07/01.

19. Falk G, Fahey T. C-reactive protein and community-acquired pneumonia in ambulatory care: Systematic review of diagnostic accuracy studies. Family practice. 2009;26(1):10-21.

20. UM Rautakorpi MS, P Carlson, J Isojarvi, P Pohja-Nylander, K Pulkki, M Makela. CRP-side test for diagnosis of pneumonia in primary care (Structured abstract). 2012(2).

21. van der Meer V, Neven AK, van den Broek PJ, Assendelft WJ. Diagnostic value of C reactive protein in infections of the lower respiratory tract: systematic review. BMJ. 2005;331(7507):26. Epub 2005/06/28.

22. Steurer J, Held U, Spaar A, Bausch B, Zoller M, Hunziker R, et al. A decision aid to rule out pneumonia and reduce unnecessary prescriptions of antibiotics in primary care patients with cough and fever. BMC medicine. 2011;9:56. Epub 2011/05/17.

23. Woloshin S, Schwartz LM. Distribution of C-Reactive Protein Values in the United States. New England Journal of Medicine. 2005;352(15):1611-3.

24. Olafsdottir IS, Gislason T, Thjodleifsson B, Olafsson I, Gislason D, Jogi R, et al. C reactive protein levels are increased in non-allergic but not allergic asthma: a multicentre epidemiological study. Thorax. 2005;60(6):451-4. Epub 2005/06/01.

25. Barnes PJ. Chronic obstructive pulmonary disease. The New England journal of medicine. 2000;343(4):269-80. Epub 2000/07/27.

26. Almirall J, Bolibar I, Balanzo X, Gonzalez CA. Risk factors for community-acquired pneumonia in adults: a population-based case-control study. Eur Respir J. 1999;13(2):349-55. Epub 1999/03/05.

27. Marrie TJ, Huang JQ. Epidemiology of community-acquired pneumonia in Edmonton, Alberta: an emergency department-based study. Canadian respiratory journal : journal of the Canadian Thoracic Society. 2005;12(3):139-42. Epub 2005/05/06.

28. Riley RD, Lambert PC, Abo-Zaid G. Meta-analysis of individual participant data: rationale, conduct, and reporting. BMJ (Clinical research ed). 2010;340:c221.

29. Stewart LA, Tierney JF. To IPD or not to IPD? Advantages and disadvantages of systematic reviews using individual patient data. Evaluation & the health professions. 2002;25(1):76-97.

30. Broeze KA, Opmeer BC, van der Veen F, Bossuyt PM, Bhattacharya S, Mol BWJ. Individual patient data meta-analysis: a promising approach for evidence synthesis in reproductive medicine. Human Reproduction Update. 2010;16(6):561-7.

31. Broeze KA, Opmeer BC, Bachmann LM, Broekmans FJ, Bossuyt PM, Coppus SF, et al. Individual patient data meta-analysis of diagnostic and prognostic studies in obstetrics, gynaecology and reproductive medicine. BMC medical research methodology. 2009;9(1):22.

32. Lyman GH, Kuderer NM. The strengths and limitations of meta-analyses based on aggregate data. BMC medical research methodology. 2005;5:14.

33. Mandell LA, Wunderink RG, Anzueto A, Bartlett JG, Campbell GD, Dean NC, et al. Infectious Diseases Society of America/American Thoracic Society consensus guidelines on the management of community-acquired pneumonia in adults. Clinical infectious diseases : an official publication of the Infectious Diseases Society of America. 2007;44 Suppl 2:S27-72. Epub 2007/02/06.

34. Haynes RB, Wilczynski NL. Optimal search strategies for retrieving scientifically strong studies of diagnosis from Medline: analytical survey. BMJ. 2004;328(7447):1040. Epub 2004/04/10.

35. Wilczynski NL, Haynes RB, Team tH, Wilczynski NL, Haynes RB, Team tH. EMBASE search strategies for identifying methodologically sound diagnostic studies for use by clinicians and researchers. BMC Medicine. 2005;3(1):7.

36. Riley RD, Simmonds MC, Look MP. Evidence synthesis combining individual patient data and aggregate data: a systematic review identified current practice and possible methods. Journal of clinical epidemiology. 2007;60(5):431-9.

37. Riley RD, Lambert PC, Staessen JA, Wang J, Gueyffier F, Thijs L, et al. Meta-analysis of continuous outcomes combining individual patient data and aggregate data. Statistics in medicine. 2008;27(11):1870-93.

38. Graffelman AW, le Cessie S, Knuistingh Neven A, Wilemssen FE, Zonderland HM, van den Broek PJ. Can history and exam alone reliably predict pneumonia? The Journal of family practice. 2007;56(6):465-70. Epub 2007/06/05.

39. Harrell FE, Jr., Lee KL, Mark DB. Multivariable prognostic models: issues in developing models, evaluating assumptions and adequacy, and measuring and reducing errors. Stat Med. 1996;15(4):361-87. Epub 1996/02/28.
