# Supplementary material for: External Validation of Prediction Models for Pneumonia in Primary Care Patients with Lower Respiratory Tract Infection: An Individual Patient Data Meta-Analysis
Source: PLoS One. 2016 Feb 26;11(2):e0149895. doi: 10.1371/journal.pone.0149895 (PMC4769284; doi:10.1371/journal.pone.0149895)
Supplement: S1 Table — (PDF) [file pone.0149895.s004.pdf]

S1 Table. Overview of methodological quality of included validation datasets according to QUADAS-2 assessment [24]**.**

Dataset	Risk of bias				Applicability concerns		
	Patient selection	*Index test*	*Reference standard*	*Flow and timing*	*Patient selection*	*Index test*	*Reference standard*
Melbye et al.	±	+	+	–	±	+	+
Hopstaken et al.	+	+	+	+	+	+	+
Flanders et al.	±	+	+	–	±	+	+
Graffelman et al.	+	+	+	+	±	+	+
Holm et al.	+	+	+	+	+	+	+
Rainer et al.	+	+	+	+	–	+	+
Steurer et al.	+	+	+	+	+	+	+
Van Vugt et al.	+	+	+	±	+	+	+
**"+"** = Low risk, "±" = Intermediate risk, "–"= High risk							
