# Supplementary material for: External Validation of Prediction Models for Pneumonia in Primary Care Patients with Lower Respiratory Tract Infection: An Individual Patient Data Meta-Analysis
Source: PLoS One. 2016 Feb 26;11(2):e0149895. doi: 10.1371/journal.pone.0149895 (PMC4769284; doi:10.1371/journal.pone.0149895)
Supplement: S2 Table — (PDF) [file pone.0149895.s005.pdf]

S2 Table. Prediction rules of included diagnostic models for pneumonia in primary care.

**Model**	**Predictors, N=**	**Predicted probability of pneumonia given by: $1/(1+e^{-y})$**
Diehr '84 [31]*	6	y= -2×Coryza + 1×Night sweats[2] + 1×Myalgia + 1×Phlegm + 2×Respiratory rate >25[3] + 2×Temperature>37.78°
Singal '89 [34]	3	y= -3.095 + 1.214×Cough[1] + 1.007×Temperature>37.78° + 0.823×Crackles
Heckerling '90 [18]	5	y= -1.705 + 0.494×Temperature >37.8°+ 0.428×Pulse>100 + 0.658×Crackles + 0.638×Diminished breath sound + 0.691×Absence of asthma
Melbye '92 [33]*	6	y= 4.7×Fever (symptom) & Dur. illness ≥1 week - 4.5×Coryza - 2.1×Sore throat +5.0×Dyspnea, very annoying[1] + 8.2×Chest pain, strong, lateral[1] + 0.9×Crackles
Hopstaken '03 [32]	3	y= -2.74 + 1.02×Dry cough[1] + 1.78×Diarrhea + 1.13×Temperature ≥38°
van Vugt '13 [19]	6	y= -3.984 + 0.446×Dyspnea + 0.698×Absence of coryza + 0.596×Diminished breath sounds + 1.404×Crackles + 0.961×Pulse>100 + 0.980×Temperature >37.8°

[1]If available (otherwise unspecified type of predictor used), [2]If available (otherwise "unspecified sweating", used), [3]In the data of Hopstaken et al. a respiratory rate >20 is used, *model does not incorporate an intercept.
