# Supplementary material for: External Validation of Prediction Models for Pneumonia in Primary Care Patients with Lower Respiratory Tract Infection: An Individual Patient Data Meta-Analysis
Source: PLoS One. 2016 Feb 26;11(2):e0149895. doi: 10.1371/journal.pone.0149895 (PMC4769284; doi:10.1371/journal.pone.0149895)
Supplement: S3 Table — (PDF) [file pone.0149895.s006.pdf]

S3 Table. In- and exclusion criteria of all model development studies and studies contributing IPD.

Study	Model	Dataset	Setting	Selection criteria	
				**Inclusion**	**Exclusion**
Diehr et al. [31]	•		ED	Adults, walk-ins, cough <1m	Pulse >160/m, temp >104°F, SBP <90 mmHg, previous consultation, pregnancy
Singal et al. [34]	•		ED	Adults, CXR request	
Heckerling et al. [18]	•		ED	Adults, fever or respir. symp., CXR performed	Pre-test (CXR) prob. Of pneumonia by physician = 0, previous consultation
Melbye et al. [33]	•	•	OHD	Adults, walk-ins between 16-21h, symp. of respir. tract or throat infection	Pregnancy, severe dyspnea
Hopstaken et al. [32]	•	•	GP	Adults, cough <1m, combination of clinical symptoms	Pregnancy, lactation, other severe disease, ABx treatment <14d, hospitalization <4w for resp. dis., previous consultation
van Vugt et al. [19]	•	•	GP	Adults, acute cough <4w, suspected of LTRI, immunocompetent	pregnancy, previous consultation, ABx <1m
Flanders et al. [35]		•	ED/AC	Adults, cough <3w	Pregnancy, syst. inflam. disorder, coexisting infect., MI, cancer, immunosupr. disorder, previous consultation
Graffelman et al. [17]		•	GP	Adults, set of clinical symptoms suggestive of LTRI	Pregnancy, cancer
Holm et al. [36]		•	GP	Adults, diagnosis of LTRI	Hospitalization <1w, illness requiring hospitalization, pregnancy
Rainer et al. [37]		•	ED	Adults, walk-ins, febrile resp. illness	

Steurer et al. [38]		•	GP, ED	Adults, new or worsened cough >24h, fever	Chronic lung disease, cough after hospitalization, pregnancy, immunocompromised
ABx=Antibiotics, AC=Ambulatory Clinic, CXR=Chest X-radiography, ED=Emergency Department, GP=General Practitioner, LRTI=Lower respiratory tract infection, MI=Myocardial infarction, OHD=Out of Hours Department, SBP=Systolic blood pressure					
