# Supplementary material for: External Validation of Prediction Models for Pneumonia in Primary Care Patients with Lower Respiratory Tract Infection: An Individual Patient Data Meta-Analysis
Source: PLoS One. 2016 Feb 26;11(2):e0149895. doi: 10.1371/journal.pone.0149895 (PMC4769284; doi:10.1371/journal.pone.0149895)
Supplement: S4 Table — (PDF) [file pone.0149895.s007.pdf]

S4 Table. Relative discriminative performance of pneumonia prediction models within datasets, measured as delta AUC. Numbers depict the difference of individual model's AUC to the average AUC of the dataset.

**Model**	**Validation dataset**							
	Melbye et al.	Hopstaken et al.	Flanders et al.	Graffelman et al.	Holm et al.	Rainer et al.	Steurer et al.	Van Vugt et al.
Van Vugt et al.	**0,06**	X	**0,14**	0,01	X	X	X	D
Heckerling et al.	-0,03	X	0,13	0,03	X	X	X	**0,04**
Diehr et al.	X	-0,02	0,00	X	X	**0,07**	X	X
Singal et al.	-0,04	**0,03**	0,05	**0,04**	NA	X	NA	0,02
Melbye et al.	D	-0,01	-0,14	-0,10	X	X	X	X
Hopstaken et al.	X	D	-0,17	0,02	X	-0,07	X	-0,06
NA = single validation within dataset, dAUC calculated, X = Model not validated in dataset due to missing predictors, D = Development dataset, dAUC not calculated.								
